# Acute necrotizing encephalopathy in an adult as a complication of H1N1 infection

**DOI:** 10.1259/bjrcr.20190028

**Published:** 2019-11-15

**Authors:** Heba S Abdelrahman, Ahmed M Safwat, Mahmoud M Alsagheir

**Affiliations:** 1 Radiology department, Faculty of medicine, Ain Shams University, Cairo, Egypt; 2 Neurology department, Saudi German Hospital, Jeddah, Saudi Arabia; 3 Anesthesia and intensive care department, Faculty of medicine, AL-Azhar University, Cairo, Egypt

## Abstract

Acute necrotizing encephalitis is one of the recognized influenza-associated encephalopathies which has a characteristic multifocal symmetric involvement of the thalami bilaterally with only very few cases were reported in adults. We present a case of a young adult female who was presented with post-H1N1 Acute Necrotizing Encephalopathy with full neurological recovery after proper clinicoradiological diagnosis and rapid treatment with steroids and intravenous immunoglobulins.

## Introduction

Influenza virus infection can cause neurological complications, despite being rare yet it is a well-known cause of morbidity and mortality all over the world. One of the recognized Influenza-associated encephalopathies is acute necrotizing encephalitis (ANE) which has a characteristic multifocal symmetric involvement of the thalami bilaterally.^1^ This disease was described for the first time by Mizuguchi et al in 1995 after reviewing the records of Japanese children who were diagnosed with encephalopathy associated with influenza virus infection.^2^ And despite reporting many cases of ANE in the pediatric population, very few cases were reported in adults.^3^

The initial presentations of the patients with this disease are seizures, vomiting, and rapidly progressive neurological decline. No preventive methods have been identified for the condition and no specific treatment with only 10% of patients completely recover.^4^

## Case presentation

A 27 year old female patient with no known comorbidities, presented with severe persistent headache, persistent vomiting, decreased level of consciousness, clonic seizures of the right side-of the face and right upper limb with incontinence.

Upon examination, the patient was confused with Glasgow Coma Scale (GCS) 8; V2 M4 E2, right facial asymmetry, hyporeflexia in both upper and lower limbs, bilateral extensor response of big toes in response to plantar stimulation (positive Babinski sign), and negative meningeal signs.

## Investigations

**Figure 1.**
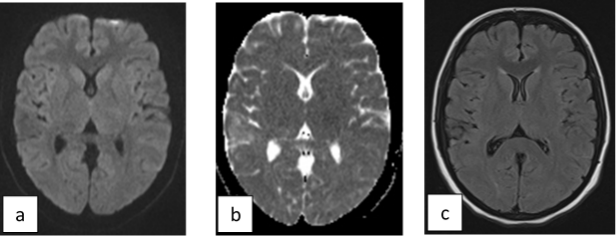
Normal MRI brain study of the patient on the first day of admission. Axial images at the level of thalami (a) axial DWI, (b) axial ADC map, (c) axial FLAIR. ADC, apparent diffusion coefficient; DWI, diffusion-weighted imaging; FLAIR, fluid-attenuated inversion-recovery.

MRI of the brain was done which was normal (Figure 1). Then laboratory work-up was performed and showed elevated white blood cells count 11.2 × 10^9^ (Neutrophils 66.2% and Lymphocytes 21.9%), with elevated inflammatory markers (Erythrocyte Sedimentation Rate 50, C-Reactive Protein 127.9, Procalcitonin 2.69).

A rapid neurological decline was noted on day two with progressive worsening of inflammatory markers (ESR 124, CRP 252.9, Procalcitonin 3.32), deranged renal and hepatic functions (Elevated AST 60 u l^−1^, Elevated urea 64 mg dl^−1^, Elevated uric acid 11.3 mg dl^−1^), and electrolyte disturbance (Elevated alkaline phosphatase 126 u l^−1^, Elevated creatinine 2.92 mg dl^−1^, Low potassium 3.4 mEq/l, Low calcium 7.7 mg dl^−1^).

An awake digital electroencephalography (EEG) performed and revealed diffuse cerebral dysfunction.

A lumbar puncture showed elevated total protein 128 mg dl^−1^, elevated chlorides 133 mEq/l, elevated cell count 10 (lymphocytes), normal glucose 108 mg dl^−1^ with no microbial growth that suggested the autoimmune process.

**Figure 2.**
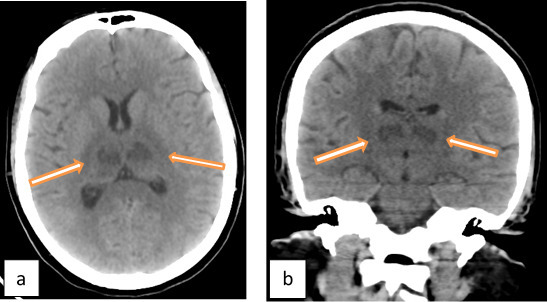
CT brain was done on day 4. (a) Axial image at the level of thalami and (b) coronal image at the level of thalami showing abnormal hypodensity with swelling at both thalami (arrows).

A CT scan of the brain was done and showed bilateral symmetric thalamic hypodensity (Figure 2).

**Figure 3.**
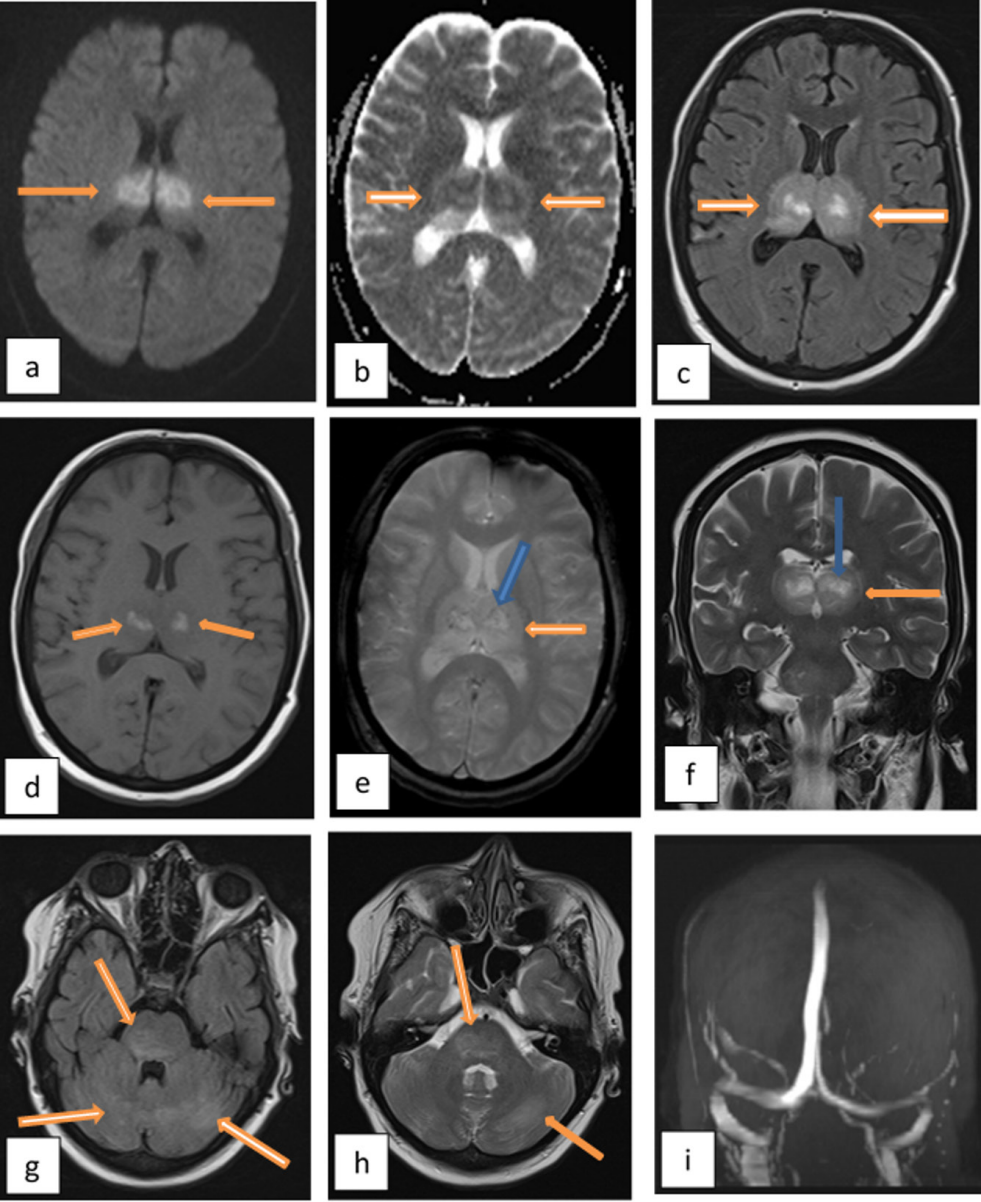
MRI brain on day 4 of admission. (a) Axial DWI and (b) ADC map at the level of thalami show areas of diffusion restriction. (c) axial FLAIR image shows edematous swollen thalami with central necrosis. (d) axial *T*_1_WI shows bright signal at the center of both thalami denoting hemorrhage. (e) axial gradient image at the same level with the mild dark blooming signal at the site of hemorrhage (blue arrow) surrounded by edema (orange arrow). (f) coronal *T*_2_WI shows the edematous thalami (orange arrow) with central necrosis (blue arrow). (g) axial FLAIR and (h) axial *T*_2_WI at the level of the posterior fossa shows the bright signal of edema at the cerebellum and pons. (i) MRV with the patent normal deep cerebral venous system. ADC, apparent diffusion coefficient; DWI, diffusion-weighted imaging; FLAIR, fluid-attenuated inversion-recovery; MRV, MR venography.

MRI brain with magnetic resonance venography (MRV) study revealed a characteristic bilateral symmetric appearance of swollen edematous thalami with central areas of necrosis and hemorrhage and ill-defined areas of edema at the cerebellar hemispheres and pones yet with normal MRV excluding hemorrhagic venous thalamic infarctions. (Figure 3)

Based upon this characteristic appearance and with the exclusion of hemorrhagic venous infarction, besides with exclusion of other differential diagnoses for encephalopathies; a diagnosis of acute necrotizing encephalitis was surfaced.

Subsequently, the connection of this rare condition in adults to viral infection was offered and the H1N1 test (Reverse transcriptase-polymerase chain reaction (RT-PCR)) was positive confirming the diagnosis of H1N1 associated acute necrotizing encephalopathy.

## Differential diagnosis

The differential diagnosis of acute onset neurological manifestations or encephalopathy which may have similar clinical, radiological, or pathological findings includes both infectious entities such as numerous viruses, bacteria, parasites and fungi and non-infectious disease entities such as acute disseminated encephalomyelitis (ADEM) after measles, antibody-associated encephalitis, which may or may not be paraneoplastic.^2,4,5^

In our case, there was a characteristic symmetric involvement of thalami bilaterally by edema and hemorrhage which points to a narrower differential diagnosis including:

1. Deep cerebral vein thrombosis which was excluded by normal MRV.
2. Osmotic demyelinating syndrome which frequently involves the thalamus, yet hemorrhage and contrast enhancement are rare.
3. Wernicke encephalopathy in which the medial part of the thalamus is the most typically involved portion. Lesions are most often symmetrical. Enhancement (especially in alcoholic patients) and/or reduced diffusion in the acute phase can be sometimes observed. Hemorrhagic lesions have been reported in catastrophic cases.
4. Reversible posterior leukoencephalopathy syndrome involves bilateral white matter in the occipital and posterior parietal lobes. However, associated involvement of grey matter and other brain are frequently seen. Yet it has certain risk factors include immunosuppressive and cytotoxic agents, hypertension, eclampsia, and metabolic abnormalities.
5. Acute disseminated encephalomyelitis in which the gadolinium-enhanced *T*_1_ imaging typically shows enhancement of all (or nearly all) the lesions.
6. Acute hemorrhagic leukoencephalitis is asymmetric in distribution, has perivascular distribution, and is associated with meningeal inflammation.
7. Acute necrotizing encephalitis despite being rare, yet it has a very characteristic symmetric involvement of both thalami.

## Treatment

Despite that the nature of this disease is not clearly recognized yet, it was proposed based upon published cases that it is an autoimmune response to the influenza virus infection, so the patient started pulse steroid therapy on Day 3 after result of cerebrospinla fluid analysis in the form of Solumedrol 1 gm + 100 ml normal saline 0.9% intravenous infusion over 1 h once daily for 5 days.

## Outcome and follow up

5 days after admission, the patient alertness increased gradually with improved mentation, she started to talk but was drowsy.

**Figure 4.**
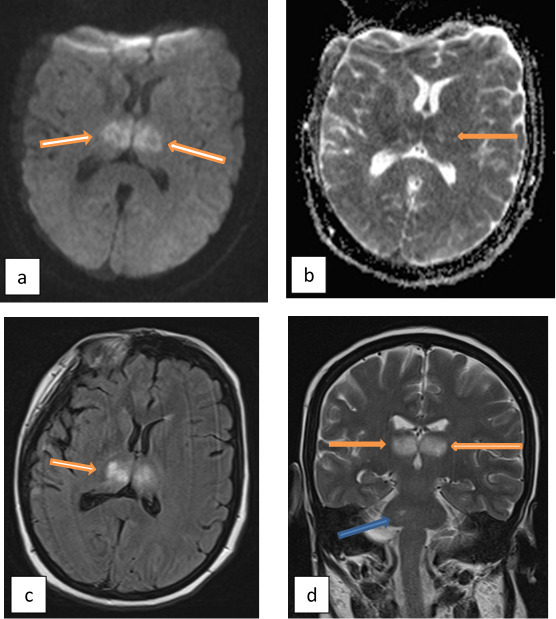
MRI brain (a) axial DWI, (b) axial ADC amp, (c) axial FLAIR at the level of thalami show a decrease in the size of the lesions and edema (arrows). (d) coronal *T*_2_WI shows a decrease in the size of the lesions at the thalami (orange arrows) and pons (blue arrow). ADC, apparent diffusion coefficient; DWI, diffusion-weighted imaging; FLAIR, fluid-attenuated inversion-recovery.

MRI brain was done and showed a decrease in the size of edematous areas especially at the thalami with a decrease in necrotic central regions. (Figure 4)

**Figure 5.**
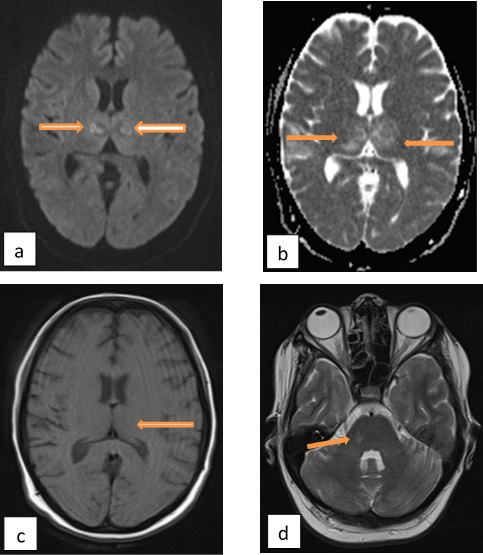
Final MRI brain was done before the discharge of the patient. (a) axial DWI and (b) axial ADC map with remarkable regression of the diffusion restriction at thalami. (c) axial *T*_1_WI at the level of thalami with the disappearance of the hemorrhagic areas. (d) axial *T*_2_WI at the posterior fossa shows disappearance of edema at the cerebellum with a marked decrease in size of edematous area at the pons. ADC, apparent diffusion coefficient; DWI, diffusion-weighted imaging

Unfortunately, the patient’s response to treatment suddenly stopped on day six and even started to deteriorate with the decline of the conscious level. So, treatment was shifted to intravenous immunoglobulins (IVIG) therapy after which the patient regained consciousness over 3 days, and within 2 weeks the patient regained full neurological function with normalization of all laboratory results and regression of brain MRI lesions. (Figure 5)

Our patient spent 27 days in intensive care unit (ICU) and upon discharge from ICU, the patient was fully conscious, Glasgow Coma Scale 15, with no sensory nor cognitive disturbances, but only complained of body pains.

## Discussion

Several neurological sequelae and complications have been connected to the influenza virus infection which includes Reye syndrome, generalized encephalopathy, seizure, aseptic meningitis, and postinfectious acute disseminated encephalomyelitis.^6^

One of these complications is influenza-associated acute necrotizing encephalopathy which has a distinct clinical and radiological feature with high mortality rates reaching 30–40%.^7^ and due to the low incidence of autopsies in these patients, the diagnosis of this specific entity was mainly depending on characteristic neuroradiologic findings.^8^

Till now, the pathogenesis of ANE is not clear, yet autopsy specimens from the patients show necrosis and petechial hemorrhages in the thalamus and tegmentum of the pons, as well as myelin pallor in the cerebral and cerebellar deep white matter. Vascular endothelial pathology and surrounding vasogenic edema without definite vascular occlusion have also been reported. These data together with the absence of influenza virus in the central nervous system (CNS) has led to the hypothesis that the inflammatory insult originates outside the central nervous system is the trigger for ANE.^9^

Other authors have hypothesized that disruption of the blood–brain barrier in the presence of systemic hypercytokinemia which is (cytokine storm) could be responsible for inducing the necrotic brain lesions that were observed in ANE.^10^

On MRI, those lesions which represent cytotoxic edema appear as hypointense areas on *T*_1_WI and hyperintense on *T*_2_WI & fluid-attenuated inversion-recovery images. This signal becomes heterogenous when hemorrhage and necrosis occur which appears as blooming in gradient images. The lesions are typically multifocal bilateral symmetric mainly involving the thalami, cerebral periventricular white matter, brainstem tegmentum, or pons and cerebellum.^5^

This characteristic appearance also reported in diffusion-weighted images with concentric areas of high signal in the periphery representing cytotoxic edema with low apparentdiffusion coefficient values and low signal in the center representing necrosis with high apparentdiffusion coefficient values.^11^ This was a typical appearance to our presented case.

It has been reported that gadolinium-contrast MRI is useful in identifying lesions at the very early stage of ANE when conventional CT, MRI, and even diffusion-weightedimaging show no abnormalities which suggest that alteration of the permeability of blood-brain barrier might be the initial step in the development of brain lesions. The contrast-enhanced MRI may, therefore, be helpful for early diagnosis to initiate the treatment as early as possible and avoid neurological sequelae of patients.^12^

It has also been concluded in the literature that the administration of steroids within 24 h after the onset of symptoms could give a better outcome in those patients provided that no brainstem lesions.^13^ which could explain the failure of therapy at the beginning in our case due to delay in the diagnosis with the subsequent start of steroid pulse therapy on Day 3.

It has been stated in the literature that the presence of hemorrhage and tissue loss in CT & MRI together with abnormal cerebrospinal fluid analysis are poor prognostic indices.^13,14^

In our case, the patient had these poor prognostic indices, yet she fully regained her neurological function despite the delay in response secondary to delay in diagnosis which could be attributed to the aggressive treatment with combined steroid and IVIG therapy.

## Learning points

1. The case illustrates the typical clinical presentation and radiological appearance of acute necrotizing encephalitis.
2. During influenza season, any case presents with unexplained central nervous system symptoms either child or adult should include in the differential diagnosis ANE.
3. No preventive methods have been identified for the condition and no specific treatment, yet it was proposed that it is an autoimmune response and rapid administration of steroids and IVIG can be of help.
